# Tinnitus assessment by means of standardized self-report questionnaires: Psychometric properties of the Tinnitus Questionnaire (TQ), the Tinnitus Handicap Inventory (THI), and their short versions in an international and multi-lingual sample

**DOI:** 10.1186/1477-7525-10-128

**Published:** 2012-10-18

**Authors:** Florian Zeman, Michael Koller, Martin Schecklmann, Berthold Langguth, Michael Landgrebe

**Affiliations:** 1 Center for Clinical Studies, University Hospital Regensburg, Regensburg, 93042, Germany; 2 Department of Psychiatry and Psychotherapy, University of Regensburg, Regensburg, Germany

**Keywords:** Tinnitus, Tinnitus handicap inventory, Tinnitus questionnaire, Tinnitus impairment questionnaire, Psychometric properties

## Abstract

**Background:**

Tinnitus research in an international context requires standardized and validated questionnaires in different languages. The aim of the present set of analyses was the reassessment of basic psychometric properties according to classical test theory of self-report instruments that are being used within the multicentre Tinnitus Research Initiative (TRI) database project.

**Methods:**

1318 patients of the TRI Database were eligible for the analyses. The basic psychometric properties reliability, validity, and sensitivity of Tinnitus Handicap Inventory (THI), Tinnitus Questionnaire (TQ) and Tinnitus Beeinträchtigungs Fragebogen (i.e., Tinnitus Impairment Questionnaire, TBF-12) were assessed by the use of Cronbach’s alpha, corrected item-total correlations, correlation coefficients and standardized response means.

**Results:**

Throughout the languages, all questionnaires showed high internal consistencies (Cronbach’s alpha > 0.79) and solid item-total correlations, as well as high correlations among themselves (around 0.8) and in combination with the self-reported tinnitus severity. However, some paradoxical correlations between individual items of the TBF-12, constructed as a shortform of the THI, and the corresponding THI-items were seen. Standardized Response Means (SRM) were low if tinnitus did not change, and between 0.3 and 1.09 for improved or worsened tinnitus complaints, indicating the sensitivity of the measures.

**Conclusions:**

All investigated instruments have high internal consistency, high convergence and discriminant validity and good change sensitivity in an unselected large multinational clinical sample and thus appear appropriate to evaluate the effects of tinnitus treatments in a cross-cultural context.

## Background

Tinnitus is an auditory sensation in the absence of any external acoustic stimuli. Tinnitus represents a frequent disorder with prevalence rates ranging between 2.4% and 20.1% [1], Chapter 5. This rather large range can be explained with the highly variable definitions used in clinical trials as well as with the different duration and nature of this condition [2].

Many people experience a “ringing in their ears” from time to time but, in the majority of cases, this phantom perception disappears spontaneously after a short time. Tinnitus symptoms are referred to as a chronic condition when lasting more than six months. Some people are able to ignore this phantom sound and do not feel severely impaired, while others suffer considerably from these symptoms. Tinnitus is also often related to depression, anxiety, and insomnia [2,3], causing severely impaired quality of life.

Several self-report instruments have been developed and validated for the assessment of tinnitus severity [4].

To promote the research and development of more efficient treatment strategies, the Tinnitus Research Initiative has set up an international database that allows the comparison of longitudinal data from patients undergoing specific, well-defined treatment interventions, either in clinical trials or in clinical routine [5]. The idea is to evaluate the effects of different treatment options (e.g. pharmacological, psychotherapeutical, auditory stimulation, brain stimulation) on an aggregate level and, eventually, to develop a decision-making tool that allows the specification of promising treatment options for patients with a given clinical condition. So far, data from eight study centers from five different countries have contributed data to this project.

The data base, which is maintained at the Tinnitus Center in Regensburg, is open for clinical studies on tinnitus that contain a standardized data set. The data set includes three patient self-report forms for assessing tinnitus symptoms, i.e. the Tinnitus Handicap Inventory (THI) [6], the Tinnitus Impairment Questionnaire (TBF-12) [7], and the Tinnitus Questionnaire (TQ) as well as its short forms Mini-TQ and TQ-12 [8].

These instruments represent validated measures; they are available in different languages and used in clinical studies around the world. Psychometric properties of the assessments instruments have been documented in various reports [6-15]. However, data about change sensitivity are only available for the THI [16], the TQ [17] and the TBF-12 [15]. The different questionnaires have only been cross-validated to a very limited extent [1]. Thus, for example, even if the TBF-12 is considered a short version of the THI [15], cross-validation data between TBF-12 and THI are not available. Moreover, replication of psychometric criteria in follow-up studies is considered important since the validity and the usefulness of self-report instruments are empirical issues that depend on a growing body of evidence [18].

Furthermore, in case of multicentre projects and questionnaires in different languages, it has to be established that such instruments work properly in every language (FDA guidance 2009). Only then analyses of multicentre projects are reliable allowing unbiased conclusions with regard to the value of therapeutic options of tinnitus symptoms.

The aim of the present set of analyses is therefore the reassessment of basic psychometric properties according to classical test theory [19,20] of self-report instruments used within the multicentre TRI database project.

## Material and methods

### Database

The data analysis was based on data of the Tinnitus Research Initiative Database. Data management was conducted according to the Data Handling Plan (TRI-DHP V05, 21.02.2011). Data analysis was conducted according to the Standard Operating Procedure (TRI-SA V01, 09.05.2011), thereby following a study-specific Statistical Analysis Plan (SAP-006) that was written according to the SAP template (TRI-SAP 006, 12.05.2011). All documents are to be found under http://database.tinnitusresearch.org/.

The data of the TRI Database stem from studies which are based on a range of various designs (randomized controlled, longitudinal, one-armed observational, cross-sectional baseline). All studies comply with a pre-specified standardized documentation set [21]. Collection of data for the TRI database was approved by the local ethics committee of the University of Regensburg, Germany.

The data set of 1 May 2011 contained a total of n=1636 patients, of which n=1318 were eligible for our analyses. The 318 excluded patients had neither baseline nor final visit values of any of the three analyzed questionnaires.

### Assessments

The Case Report Form (CRF) of the TRI database contains different types of questionnaires (medical history, audiological examinations, tinnitus related questionnaires, depression, and quality of life) in different languages (German, Dutch, Portuguese, and Spanish); all questionnaires were collected in a standardized manner [21].

The questionnaires relevant for this analysis were filled in by the patients at each study visit in the following order: 1) Tinnitus Handicap Inventory (THI), 2) Tinnitus Questionnaire (TQ), 3) Tinnitus Beeinträchtigungs-Fragebogen (i.e., Tinnitus Impairment Questionnaire, TBF-12), 4) Tinnitus Severity Scale, and 5) Patient Global Impression-Change (PGI-C).

The most common of the above questionnaires is the THI [6], which has already been translated into and validated in different languages, such as Chinese [22], Portuguese [9], Danish [13], German [12], French [23], and Italian [24,25]. Its primary use is the stratification of patients with tinnitus according to the impact of tinnitus symptoms in daily life [11]. Moreover, the THI has been often used as a primary outcome measurement for testing the efficacy of therapeutic interventions, even if the questionnaire has not been primarily developed for this purpose.

The TBF-12, developed by Greimel et al. [7], is a short version of the THI that includes 12 instead of 25 questions. However, a few questions are formulated in a slightly different manner, and the responses are “never ─ sometimes ─ often”. In contrast, responses of the THI are “yes ─ sometimes ─ no” in an opposite order. To further complicate matters, the TBF-12 has recently been renamed as THI-12 [15].

The TQ is a self-report measure of tinnitus-related distress as perceived by patients [8]. This questionnaire has been translated into and validated in different languages, into German [26], Dutch, French [10], and Chinese [27]. In the current study, the sum score was calculated according to the validation of the TQ in the German language, which was based on 40 out of all 52 items [28]; items nos. 5 and 20 were counted double [26]. The TQ, the most widely used questionnaire in German-speaking areas, has also been applied as a primary outcome measurement in various clinical trials [5]. Because the 52 questions of the TQ are a rather high number to be filled in by patients during every visit, two shorter versions, the TQ 12 and the Mini-TQ, have been developed. The TQ 12, developed by Hiller and Goebel [29] according to an optimal combination of high item-total correlations, reliability, and sensitivity in assessing changes, consists of 12 items that correspond to items nos. 5, 9, 17, 24, 28, 34, 35, 36, 39, 43, 47, and 48 of the TQ. The Mini-TQ consists of 10 items, corresponding to items nos. 4, 11, 15, 17, 24, 34, 35, 39, 47, and 48. The two short versions are not an extra part of the TRI Case Report Form but can be easily extracted and calculated.

Both the Tinnitus Severity Scale and the Patient Global Impression–Change (PGI-C) consist of only one question to measure the subjective perception of patients about their tinnitus severity at the time and the change of tinnitus complaints over time. The Tinnitus Severity Scale asks patients how much of a problem tinnitus symptoms are at the time and provides the following response options: not a problem, a small problem, a moderate problem, a big problem, or a very big problem. A general review about self-rating scales can be found at Wewers et al. [30]. The PGI-C asks patients to rate the total abatement of their tinnitus complaints compared with the time before treatment and provides the following response options: very much better, much better, minimally better, no change, minimally worse, much worse, and very much worse.

For each questionnaire, all subscales, number of items, response scales, and score ranges are summarized in Table 1. Since patients from some of the centers have not filled in every questionnaire and some patients have had only baseline data, the number of complete questionnaires for each language and each visit are shown in Table 2. The median time interval between baseline and final visit assessments is 3.0 months (IQR 2.4 to 4.5 months).

**Table 1:** Description of questionnaires

**Questionnaire**	**(sub)scales**	**No of items**	**Response scale**	**Range of score**
**THI**	Total	25	0/2/4 (yes/sometimes/no)	0-100
TBF12*	Total	12	0/1/2 (never/sometimes/often)	0-24
**TQ**	Total	52 (40**)	0/1/2 (true/partly true/not true)	0-84
	Emotional distress	12	0/1/2 (true/partly true/not true)	0-24
	Cognitive distress	8	0/1/2 (true/partly true/not true)	0-16
	Intrusiveness	8	0/1/2 (true/partly true/not true)	0-16
	Auditory perceptual difficulties	7	0/1/2 (true/partly true/not true)	0-14
	Sleep disturbances	4	0/1/2 (true/partly true/not true)	0-8
	Somatic complaints	3	0/1/2 (true/partly true/not true)	0-6
Mini-TQ***	Total	10	0/1/2 (true/partly true/not true)	0-20
TQ 12***	Total	12	0/1/2 (true/partly true/not true)	0-24

* modified subset of THI items (Greimel et al.).

** 40 out of 52 items are used for TQ total score, and items 5 and 20 are counted double.

*** extracted from the TQ.

**Table 2:** Number of questionnaires

**Questionnaire**	**Total**	**Germany (German)**	**Belgium (Dutch)**	**Brazil (Portuguese)**	**Argentina (Spanish)**
**THI totalscore**					
- Baseline	1292	1127	34	98	33
- Final Visit	350	237	19	78	16
**TBF12 totalscore**					
- Baseline	685	534	35	83	33
- Final Visit	341	238	19	71	13
TQ* totalscore					
- Baseline	1108	1108	0	0	0
- Final Visit	309	309	0	0	0
**Tinnitus severity**					
- Baseline	1139	995	29	82	33
- Final Visit	364	263	14	71	16
**PGI-C**					
- Final Visit	429	328	18	79	4

* same for Mini-TQ and TQ 12.

### Statistical analysis

Patient characteristics are summarized as median values and interquartile ranges (first to third quartiles) for continuous variables as well as frequency counts and percentages for categorical data. Statistical analyses were done with IBM SPSS Statistics 19.0.

### Reliability

To assess *internal consistency*, Cronbach’s alpha and the corrected item-total correlations were calculated with baseline and final visit data.

### Validity

*Convergent validity* is the degree to which all tinnitus-related questionnaires measure the same underlying construct. To assess convergent validity, Pearson’s correlation was computed to measure the association between the total scores of all tinnitus-related questionnaires. To analyze relations between the TBF12 and the corresponding questions of the THI, we used cross tabulations as well as Spearman’s rank correlation coefficient for each question.

*Known group differences* (*discriminant validity*) involve the assessment of a tinnitus questionnaires’ ability to distinguish between different levels of tinnitus severity. In this analysis, scores of each questionnaire were compared to tinnitus-related severity (not a problem, a small problem, a moderate problem, a big problem, a very big problem) rated by patients themselves. To test if higher scores of each questionnaire are linearly related to a higher level of patient-self-reported severity, we conducted the Spearman's rank correlation coefficient.

### Sensitivity

To assess change sensitivity, we used the PGI-C to identify whether patients changed over time. Because of the small sample sizes in some PGI-C categories, the data were pooled into the categories ‘improved’ (PGI-C values 1–3), ‘unchanged’ (PGI-C value 4), and ‘worsened’ (PGI-C values 5–7) to yield a sufficient number of cases in each category. To assess the magnitude of the difference in scores between patients who improved, unchanged, or worsened, we calculated standardized response means (SRMs) by dividing the mean score changes by the standard deviation (SD) of the change [31]. We compared the SRMs against Cohen’s rough rule of thumb for interpreting the magnitude of the mean differences of each questionnaire, which suggests that a change of 0.20 represents a small change, 0.50 a moderate change, and >0.80 a large change [31].

## Results

### Patient characteristics

Out of n=1318 patients, 1149 were from Germany, 38 from Belgium, 98 from Brazil, and 33 from Argentina. Patients were aged between 10 and 89 years (median 53.2 years, interquartile range [IQR] 44.7 to 62.3 years). Tinnitus duration was between 1 month and 52 years (median 5.2 years, IQR 1.6 to 12.2 years). Baseline characteristics and other tinnitus-related information are presented in Table 3. To assess if patients’ heterogeneity in age and tinnitus duration has influence on tinnitus severity and thus is potentially confounding the results, we analyzed the correlations between both variables and all questionnaires. The correlation coefficients according to Spearman’s rho are very low (ranging between −0.03 and 0.16), hence in our sample neither age nor tinnitus duration did influence tinnitus severity.

**Table 3:** Patient characteristics (n=1318)

**Age (years), median (IQR)**	**53.2 (44.7; 62.3)**	
**Tinnitus duration (years), median (IQR)**	**5.2 (1.6; 12.2)**	
**Sex** (N, %)		
Male	844	(64.0%)
Female	474	(36.0%)
**Country (language)** (N,%)		
Germany (German)	1149	(87.2%)
Belgium (Dutch)	38	(2.9%)
Brazil (Portuguese)	98	(7.4%)
Argentina (Spanish)	33	(2.5%)
**Laterality** (N, %^*^)		
Right	391	(29.7%)
Left	504	(38.2%)
Both sides/inner head	401	(30.4%)
**Tinnitus severity at baseline on the basis of the THI** (N, %^*^)		
Slight	129	(9.8%)
Mild	344	(26.1%)
Moderate	361	(27.4%)
Severe	307	(23.3%)
Catastrophic	151	(11.5%)
**Etiology** (N, %^**^)		
Noise trauma	238	(18.1%)
Injury of cervical spine	174	(13.2%)
Change of hearing	327	(24.8%)
Stress	565	(42.9%)
Head injury	155	(11.8%)
Other	573	(43.4%)
**Type of treatment** (N, %)		
Pharmacological treatment	237	(18.0%)
Transcranial direct current stimulation	33	(2.5%)
Transcutaneous vagus nerve stimulation	24	(1.8%)
Repetitive transcranial magnetic stimulation	260	(19.7%)
Other	37	(2.8%)
None (only baseline)	727	(55.2%)

IQR: interquartile range,

^*^ do not add up to 100% or n=1318 due to occasional missing values.

^**^ do not add up to 100% or n=1318 due to multiple answers.

### Reliability

We calculated the internal consistencies of all questionnaires according to the different languages as well as the item-total correlation coefficients. Cronbach’s alpha varied between 0.79 and 0.96 indicating a high internal consistency for all questionnaires in all languages (Table 4).

**Table 4:** Cronbachs alpha

**Questionnaire**	**Total**	**Germany (German)**	**Belgium (Dutch)**	**Brazil (Portuguese)**	**Argentina (Spanish)**
**THI**					
- Baseline	0.92	0.93	0.93	0.92	0.95
- Final Visit	0.94	0.94	0.93	0.95	0.93
**TBF12**					
- Baseline	0.88	0.88	0.89	0.84	0.92
- Final Visit	0.90	0.89	0.90	0.92	0.79
**TQ**					
- Baseline	0.95	0.95	-	-	-
- Final Visit	0.95	0.95	-	-	-
**Mini-TQ**					
- Baseline	0.87	0.87	-	-	-
- Final Visit	0.88	0.88	-	-	-
**TQ 12**					
- Baseline	0.87	0.87	-	-	-
- Final Visit	0.87	0.87	-	-	-

The corrected item-total correlations for the THI items varied widely from −0.19 to 0.85. Question no. 24 seemed to have a low (<0.3) overall correlation; however, some questions only had a low correlation in specific languages, for instance, questions nos. 8, 11, and 19 in Dutch, 2 and 8 in Portuguese, and 9, 13, and 17 in Spanish.

For the TBF-12, the corrected item-total correlations mainly ranged between 0.4 and 0.6, and only questions nos. 5, 7, and 8 showed correlations of <0.3 for the Spanish version, which might be due to the small sample size.

The corrected item-total correlations for the Tinnitus Questionnaire for baseline and last visit had a narrow range from 0.344 to 0.695, and none of the questions showed a correlation below 0.3.

Summaries for all corrected item-total correlations coefficients for all questionnaires can be found in the Additional file 1 available in the online version of the journal.

### Validity

Correlations between tinnitus-related questionnaires were very high (around 0.8) except for the Spanish version; THI vs. TBF12 at last visit was r=0.43, which was not significant because of the small sample size. All correlations of all questionnaires for each language are summarized in Table 5.

**Table 5:** Correlations between questionnaires

**Questionnaire**		**THI**		**TBF12**		**TQ**		**TQ Mini**	
		**Baseline**	**Final**	**Baseline**	**Final**	**Baseline**	**Final**	**Baseline**	**Final**
**TBF12**	Total	0.84 (n=678)	0.86 (n=339)	1					
	German	0.87 (n=528)	0.89 (n=236)						
	Dutch	0.64 (n=34)	0.90 (n=19)						
	Portuguese	0.79 (n=83)	0.78 (n=71)						
	Spanish	0.79 (n=33)	0.43* (n=13)						
**TQ**	Total/ German	0.87 (n=1089)	0.90 (n=217)	0.83 (n=503)	0.85 (n=218)	1			
**Mini-TQ**	Total/ German	0.87 (n=1052)	0.90 (n=216)	0.82 (n=490)	0.87 (n=217)	0.93 (n=1071)	0.94 (n=307)	1	
**TQ 12**	Total/ German	0.87 (n=1056)	0.89 (n=214)	0.81 (n=490)	0.85 (n=215)	0.94 (n=1074)	0.95 (n=305)	0.96 (n=1063)	0.96 (n=305)

All correlations are significant with p<.05 except the ones marked by *.

We evaluated the construct validity of the questionnaires by examining their respective correlations and mean values with the subjective ratings on tinnitus severity. Tables 6 and 7 summarize the THI, TBF12, TQ, Mini-TQ, and TQ-12 scores according to the categories of patient-perceived severity (not a problem, a small problem, a moderate problem, a big problem, a very big problem) at baseline and final visit. The THI correlated highly with the reported degree of severity in an interval between 0.57 and 0.70 for each language. The two outliers with r=0.92 and r=0.21 can be explained by the small number of patients in some categories of the tinnitus severity scale. The TBF12 is also correlated with tinnitus severity (0.45 ≤ r ≤ 0.62) and also showed two outliers because of small sample sizes. The TQ as well as the sub scores Mini-TQ and TQ-12 correlated highly between 0.65 and 0.68.

**Table 6:** Known group differences with regard to Tinnitus severity at baseline

**Tinnitus severity at baseline**	**Not a problem**		**A small problem**		**A moderate problem**		**A big problem**		**A very big problem**		**Correlation (Spearman)**
**Questionnaire**	**n**	**Mean (SD)**	**n**	**Mean (SD)**	**n**	**Mean (SD)**	**n**	**Mean (SD)**	**n**	**Mean (SD)**	**r**
**THI**											
- total	17	20.0 (11.5)	149	26.5 (16.5)	425	37.8 (17.6)	419	58.0 (18.6)	113	74.1 (15.8)	0.64**
- German	16	20.8 (11.5)	136	26.4 (16.9)	366	37.9 (17.7)	378	58.1 (18.4)	87	73.4 (15.8)	0.63**
- Dutch	0	-	0	-	9	37.3 (14.5)	6	64.7 (19.2)	10	75.0 (15.3)	0.70**
- Portuguese	1	8.0 (−)	12	28.0 (12.6)	36	39.3 (16.6)	21	55.6 (17.8)	12	76.5 (16.9)	0.67**
- Spanish	0	-	1	14.0 (−)	14	32.1 (19.9)	14	54.6 (14.9)	4	80.5 (17.1)	0.63**
**TBF12**											
- total	5	4.6 (4.7)	65	7.4 (4.1)	236	10.0 (4.1)	234	14.2 (4.6)	70	18.7 (4.0)	0.61**
- German	5	4.6 (4.7)	54	7.7 (4.2)	179	10.3 (4.0)	196	14.5 (4.5)	48	19.0 (3.8)	0.61**
- Dutch	0	-	0	-	10	10.2 (4.9)	6	15.7 (4.0)	10	18.1 (4.7)	0.62**
- Portuguese	0	-	10	6.0 (3.7)	33	9.1 (4.1)	18	12.0 (4.0)	8	17.5 (3.8)	0.58**
- Spanish	0	-	1	5.0 (−)	14	8.4 (4.6)	14	12.2 (5.9)	4	19.0 (5.9)	0.51**
**TQ**											
- German	14	17.6 (11.2)	135	21.9 (11.3)	355	33.4 (13.9)	373	49.6 (13.3)	84	61.6 (12.1)	0.68**
**Mini-TQ**											
- German	13	5.6 (4.5)	128	5.8 (3.4)	344	8.9 (4.0)	363	13.5 (3.6)	80	16.4 (3.0)	0.67**
**TQ 12**											
- German	13	5.5 (4.3)	132	7.5 (4.2)	342	10.7 (4.7)	366	16.0 (4.3)	81	19.3 (3.6)	0.65**

* p<0.05; **p<0.001.

**Table 7:** Known group differences with regard to Tinnitus severity at final visit

**Tinnitus severity at final visit**	**Not a problem**		**A small problem**		**A moderate problem**		**A big problem**		**A very big problem**		**Correlation (Spearman)**
**Questionnaire**	**n**	**Mean (SD)**	**n**	**Mean (SD)**	**n**	**r**	**n**	**Mean (SD)**	**n**	**Mean (SD)**	**r**
**THI**											
- total	6	1.3 (1.6)	43	19.9 (16.4)	125	34.7 (18.8)	82	54.6 (18.0)	18	67.3 (19.5)	0.65**
- German	1	0.0 (−)	16	19.4 (14.2)	79	37.0 (19.2)	64	55.0 (17.5)	14	68.3 (19.1)	0.60**
- Dutch	0	-	0	-	6	30.3 (11.8)	6	55.3 (8.8)	2	82.0 (14.1)	0.92**
- Portuguese	5	1.6 (1.7)	27	20.2 (17.8)	27	27.2 (15.2)	9	53.1 (26.4)	2	46.0 (11.3)	0.57**
- Spanish	0	-	0	-	13	38.2 (22.8)	3	50.0 (21.1)	0	-	0.21
**TBF12**											
- total	5	0.2 (0.4)	41	6.6 (5.2)	123	9.4 (4.6)	79	14.6 (4.4)	18	18.8 (3.7)	0.62**
- German	1	0.0 (−)	15	7.2 (3.6)	81	10.5 (4.2)	64	15.1 (4.2)	14	18.7 (3.9)	0.61**
- Dutch	0	-	0	-	6	7.7 (5.7)	6	13.2 (2.0)	2	21.5 (2.1)	0.75**
- Portuguese	4	0.3 (0.5)	26	6.3 (6.0)	25	6.6 (4.6)	7	12.9 (4.7)	2	16.5 (0.7)	0.45**
- Spanish	0	-	0	-	11	8.6 (4.3)	2	6.0 (1.4)	0	-	−0.23
**TQ**											
- German	3	13.0 (13.5)	36	21.0 (13.6)	105	33.4 (14.3)	84	50.9 (12.5)	20	60.9 (15.7)	0.67**
**Mini-TQ**											
- German	3	3.3 (4.2)	35	5.4 (3.7)	105	9.1 (4.1)	83	13.7 (3.2)	20	16.1 (3.6)	0.67**
**TQ 12**											
- German	3	4.3 (5.1)	34	6.6 (4.5)	105	10.8 (4.8)	82	15.9 (3.7)	20	18.7 (4.7)	0.65**

* p<0.05; **p<0.001.

### Cross-validation of THI and TBF-12

Because the TBF-12 consists of 12 items of the THI with a slightly different wording of some of the questions as well as the answer options (TBF12: never ─ sometimes ─ often vs. THI: yes ─ sometimes ─ no), we compared the given answers of both questionnaires. The calculated correlations over all languages of r=0.49 to r=0.85 were lower than expected given that the TBF-12 had been developed as a short version of the THI (Table 8). The cross tabulations showed that many patients (up to 50% for some questions) who had answered a question with “never” or “often” in the TBF12 used “sometimes” for the same question in the THI and vice versa. A minority of patients (9 to 40) even gave opposing answers for the corresponding questions of the two questionnaires. The cross table for THI question no. 23 (“Do you feel that you can no longer cope with your tinnitus?”) and the corresponding TBF-12 question no. 12 (“Do you feel that you can’t cope with your tinnitus?”) is shown as an example in Table 9. An analysis of those patients who gave opposing answers did not show any systematic error patterns, such as giving the same answers (e.g. only “yes”) throughout the questionnaire or confusing “yes” and “no” (data not shown).

**Table 8:** Correlations between TBF12 and corresponding THI questions at baseline

**THI/TBF12**	**Q1/Q1**	**Q2/Q2**	**Q3/Q3**	**Q8/Q4**	**Q9/Q5**	**Q10/Q6**	**Q13/Q7**	**Q15/Q8**	**Q17/Q9**	**Q18/Q10**	**Q22/Q11**	**Q23/Q12**	**Total**
Total (n=667)	0.67	0.72	0.47	0.49	0.76	0.64	0.72	0.76	0.75	0.55	0.68	0.50	0.85
German (n=521)	0.67	0.76	0.50	0.53	0.74	0.67	0.72	0.77	0.76	0.60	0.74	0.55	0.88
Dutch (n=34)	0.74	0.83	0.64	0.62	0.70	0.73	0.67	0.77	0.81	0.55	0.61	0.51	0.70
Portuguese (n=79)	0.57	0.51	0.53	0.43	0.74	0.58	0.67	0.70	0.69	0.38	0.29	0.27	0.81
Spanish (n=33)	0.76	0.66	0.44	0.14 (n.s.)	0.67	0.47	0.64	0.63	0.57	0.52	0.83	0.51	0.78

**Table 9:** Cross table for THI question 23 and the corresponding TBF-12 question 12 at baseline

	TBF-12, item 12**			
THI, item 23*	**Never**	**Sometimes**	**Often**	**Total**
No	103	154	25	282
Sometimes	13	166	87	266
Yes	13	29	77	119
Total	129	349	189	667

* Q: “Do you feel that you can no longer cope with your tinnitus?”

** Q: “Do you feel that you can’t cope with your tinnitus?”

### Sensitivity

The SRMs of patients whose tinnitus complaints improved according to the PGI-C ranged between 0.80 and 1.09 for the THI and between 0.59 and 1.00 for the TBF12. For the German versions of the TQ, Mini-TQ, and TQ-12 the SRMs were calculated to be 1.04, 0.95, and 0.94, respectively. Patients rating themselves as unchanged showed slightly positive mean score changes and the SRMs were low ranging between 0.06 to 0.26, except for the Dutch and Portuguese versions of the THI with SRMs of 0.45 and 1.05 respectively. The SRMs of patients who reported worsening of tinnitus complaints could only be calculated for the German versions of the questionnaires because of the small sample sizes of the Dutch, Portuguese, and Spanish versions. In this category, mean changes were negative, and the SRMs varied between 0.29 and 0.44. All SRMs are summarized in Table 10.

**Table 10:** Change sensitivity

** PGI-C**	**Improved**			**Unchanged**			**Worsened**		
**Questionnaire**	**n**	**Mean change (last - base)**	**SRM**	**n**	**Mean change (last - base)**	**SRM**	**n**	**Mean change (last - base)**	**SRM**
**THI**									
- total	126	14.46	0.89	136	3.25	0.26	61	−3.25	0.30
- German	61	9.57	0.80	109	1.50	0.13	55	−3.67	0.35
- Dutch	5	8.00	0.93	7	3.71	0.45	4	-	-
- Portuguese	58	20.34	1.09	19	13.37	1.05	1	-	-
- Spanish	2	-	-	1	-	-	1	-	-
**TBF12**									
- total	116	2.74	0.71	132	0.23	0.08	61	−1.34	0.36
- German	59	2.05	0.59	111	0.18	0.06	55	−1.56	0.44
- Dutch	6	2.00	1.00	7	0.71	0.21	4	-	-
- Portuguese	50	3.62	0.84	13	0.46	0.11	1	-	-
- Spanish	1	-	-	1	-	-	1	-	-
**TQ**									
- German	86	8.72	1.04	138	0.96	0.11	71	−3.89	0.38
**Mini-TQ**									
- German	85	2.36	0.95	137	0.29	0.11	70	−0.80	0.30
**TQ 12**									
- German	83	2.83	0.94	135	0.27	0.09	70	−1.0	0.29

PGI-C: Patient Global Impression-Change categorized into 3 groups (improved, unchanged and worsened).

Mean change (last-base): Positive values mean improvement of tinnitus complaints.

SRM: Absolute values of Standardized Response Mean (mean score changes divided by the standard deviation of the change, SD data not shown). Classification according to Cohen: ≈0.2 small, ≈0.5 moderate, >0.8 large change.

For group sizes <5 neither mean change nor SRM was calculated.

## Discussion

Tinnitus Questionnaires such as the THI have been translated into several different languages, including German, Dutch, Spanish, Portuguese, Turkish, Danish, and Chinese. Thus, a psychometric validation of these types of questionnaires for each language adaption is important as well as a comparison of the results, and an examination of any differences that may exist, either in the translation process or in cultural matters. In the present study, we compared the German, Dutch, Spanish, and Portuguese versions of the THI and the TBF-12 (a modified subset of the THI) and, additionally, the German Version of the TQ with its subsets Mini-TQ and TQ 12. Data for the analyses were derived from the TRI Database, standing for a highly standardized and controlled data collection of international clinical studies on tinnitus.

In the first step, we assessed the reliability of the questionnaires throughout all languages and then for each language separately. A well-known measurement parameter for internal consistency is Cronbach’s Alpha, which means that all test items measure the same construct. As a rough rule of thumb, values greater than 0.7 show good internal consistencies and values greater than 0.9 very high internal consistencies [32]. In the present study, we found high values between 0.79 and 0.95, indicating very good internal consistency throughout the languages for all questionnaires. Interestingly, the difference in Cronbachs alpha between the longer and shorter questionnaires is only about 0.07 or 0.08 points. This increase in alpha is not substantial and simply reflects an artificial inflation due to the higher number of items [33]. Thus the longer versions don’t show more reliability than the shorter ones.

To check if each item is consistent with the average direction of the other items, an item-total correlation test was done for each questionnaire in each language. If the correlation (calculated with Pearson’s correlation coefficient) between one single item and the total score without this item correlates low (values less than 0.3) [34], then the item does not measure the same construct as the others. For the THI questionnaire, item no 2 showed relatively low item-total correlations at baseline, which is in accordance with previous findings reported by Newman et al. [6]. Interestingly, item-total correlations were considerably higher at final visit. Unexpectedly, item 24 showed low item-total correlations (< .30) in the whole sample and also in the German and Dutch subsamples. Nevertheless, due to its high-content validity of the (“tinnitus gets worse under stress”), the item should be retained (cf. Newman et al.). The lowest item-total correlations at baseline and at final visit (<. 2 and < −.2) occurred with respect to item no 19, but only in Dutch. At this stage it is unclear whether there is a problem with the translation or the finding is simply an artifact due to the relatively low sample size. These data indicated that *worsening under stress* (item 24), and *hearing difficulties due to tinnitus loudness* (item 2) were only lowly correlated with tinnitus handicap. The items of the TBF12 as well of the TQ generally showed good item/total correlations and thus seemed to measure the same construct.

Since all questionnaires had been designed to measure the strength of patients’ tinnitus complaints, we calculated the correlations between the questionnaires. A low correlation would indicate differences between the constructs of the questionnaires measure. Although we found high correlations among all questionnaires, the correlation between THI and TBF12 was surprisingly low, given that the TBF12 is a modified subset of the THI. To explore potential reasons for this relatively low correlation, we correlated all items of the TBF-12 with the corresponding items of the THI. Since the questions had the same content in corresponding items, high correlations (>0.8) were expected for this analysis. Instead, correlations ranged between 0.5 and 0.7 and some were even lower than 0.5 (see Table 8). The most likely explanation for these low correlations is the differences in the answer scales of the TBF-12 and the THI (TBF-12: “never ─ sometimes ─ often”; THI: “yes ─ sometimes ─ no”) and the slightly different wording of some of the questions. Table 9 illustrates in detail how much the response to the same question varied in the two questionnaires. The different order of the answer options cannot entirely explain the variability, since no systematic error pattern could be found for patients giving opposing answers. Since all patients had to complete all questionnaires at each assessment, the inconsistencies could also be due to lack of concentration or inattention. Nevertheless, our findings indicate that already small changes in the structure of questionnaire items may have substantial consequences. Moreover, our results indicate relevant differences between the answers to the 12 items of the TBF-12 and the corresponding items of the THI. Therefore, the TBF-12 should rather be regarded as a separate questionnaire than as a short version of the THI.

Another important aspect of the validity of questionnaires is whether the total score distinguishes between known groups of patients, e.g. different grades of tinnitus severity. In this context, we could show a clear linear relation between a global estimation of tinnitus severity (the answer to the question: “How much of a problem is your tinnitus?”) and the corresponding scores for all questionnaires in all languages except for the Spanish version. The low correlation in the Spanish version was related to the very small sample size in this group.

Although most tinnitus questionnaires were designed to measure tinnitus complaints at the time but not the change of tinnitus over time, they are often used for detecting treatment-related improvements and also serve as a primary endpoint in clinical trials. First analyses for change sensitivity can be found for the THI [16], the TBF-12 [15] and the TQ [17], but not for the Mini-TQ, and the TQ 12. For use as an outcome parameter in clinical trials, an important point in the analysis of questionnaires is change sensitivity, indicating the potential of a questionnaire to map the change of tinnitus over time. We could show that all questionnaires have very good sensitivity when tinnitus complaints decrease or increase. For unchanged tinnitus complaints, the difference in the scores between final visit and baseline should ideally stay constant, resulting in a very low SRM. In our analysis, the Dutch and the Portuguese versions of the THI had relatively high SRM values for unchanged tinnitus complaints, whereas all other questionnaires proved to be robust. Since sample sizes for both, the Dutch and the Portuguese version are quite low in the unchanged group (n<20), the observed effects may be idiosyncratic. Further studies are needed to confirm or revise the calculated SRMs, before further conclusions can be drawn. Surprisingly, the subsets Mini-TQ and TQ 12 of the TQ showed the best results despite the small number of items, indicating their usefulness as outcome measures. However, all our conclusions with respect to the Mini-TQ and the TQ 12 are preliminary since they were derived from an analysis of extracted items of the TQ. We cannot exclude the possibility that completion of the short versions may result in different results.

## Conclusion

The present analysis showed that the THI performs comparably well across several countries and languages. The TBF-12 performed equally well with respect to its summary score, although surprisingly low correlations were found between several individual items assessing the same content in both questionnaires. Therefore, caution is warranted when the TBF-12 is regarded as a short form of the THI. The TQ, which was only investigated in German, also showed satisfying psychometric results, which were equally good in the long forms and the short forms. In summary, all investigated instruments had high internal consistency, high convergent validity, good change sensitivity, and discriminated between patients with different tinnitus severity in an unselected large multinational clinical sample. Thus, the questionnaires are appropriate to evaluate the effects of tinnitus treatments in a cross-cultural context.

## Competing interests

The authors declare that they have no competing interests.

## Authors’ contributions

FZ and MK conceptualized the project and developed the statistical analysis plan (SAP). The SAP was reviewed by all authors. MS, BL, ML and the members of the TRI database study contributed to data collection. FZ analyzed the data and drafted the manuscript. All authors contributed to the interpretation of data and to the final version of the manuscript. All authors read and approved the final manuscript.

## Authors’ information

TRI database study group: Ricardo Figueiredo, Andreia Aazevedo, Marcello Rates, Carolina Binetti, Ana Belen Elgoyhen, Dirk De Ridder, Sven Vanneste, Susanne Staudinger, Elmar Frank, Peter M Kreuzer, Timm B Pöppl, Astrid Lehner.

## Supplementary Material

Additional file 1 **Corrected item/total correlation for THI.** Corrected item/total correlation for TBF12. Corrected item/total correlation for TQ. Click here for file
