# Supplementary material for: Tinnitus assessment by means of standardized self-report questionnaires: Psychometric properties of the Tinnitus Questionnaire (TQ), the Tinnitus Handicap Inventory (THI), and their short versions in an international and multi-lingual sample
Source: Health Qual Life Outcomes. 2012 Oct 18;10:128. doi: 10.1186/1477-7525-10-128 (PMC3541124; doi:10.1186/1477-7525-10-128)
Supplement: Additional file 1 — Corrected item/total correlation for THI. Corrected item/total correlation for TBF12. Corrected item/total correlation for TQ. [file 1477-7525-10-128-S1.docx]

Corrected item/total correlation for THI

| **Corrected Item/total correlation of the THI questions** | | | | | | | | | | | |  |
| --- | --- | --- | --- | --- | --- | --- | --- | --- | --- | --- | --- | --- |
| **No** | **Question** | **Total** | | **German** | | **Dutch** | | **Portuguese** | | **Spanish** | | |
|  |  | **BL**  **(n=1199)** | **FV**  **(n=318)** | **BL**  **(n=1047)** | **FV**  **(n=214)** | **BL**  **(n=34)** | **FV**  **(n=18)** | **BL**  **(n=87)** | **FV**  **(n=70)** | **BL**  **(n=31)** | **FV**  **(n=16)** | |
| 1 | Because of your tinnitus is it difficult to concentrate? | .626 | .702 | .626 | .685 | .570 | .730 | .587 | .651 | .808 | .789 | |
| 2 | Does the loudness of your tinnitus make it difficult for you to hear people? | .308 | .492 | .305 | .489 | .349 | .637 | .284 | .363 | .443 | .757 | |
| 3 | Does your tinnitus make you angry? | .579 | .585 | .586 | .580 | .671 | .586 | .524 | .773 | .635 | .578 | |
| 4 | Does your tinnitus make you feel confused | .538 | .580 | .537 | .682 | .795 | .821 | .547 | .670 | .572 | .345 | |
| 5 | Because of your tinnitus do you feel desperate? | .725 | .735 | .733 | .734 | .643 | .618 | .728 | .715 | .637 | .585 | |
| 6 | Do you complain a great deal about your tinnitus? | .497 | .525 | .507 | .517 | .355 | .369 | .547 | .706 | .639 | .685 | |
| 7 | Because of your tinnitus do you have trouble falling to sleep at night? | .496 | .483 | .496 | .404 | .398 | .527 | .509 | .533 | .501 | .533 | |
| 8 | Do you feel that you cannot escape your tinnitus? | .485 | .548 | .510 | .491 | .402 | .210 | .242 | .597 | .569 | .572 | |
| 9 | Does your tinnitus interfere with your ability to enjoy social activities (such as going out to dinner, to the movies)? | .591 | .672 | .604 | .691 | .405 | .433 | .659 | .648 | .451 | .283 | |
| 10 | Because of your tinnitus do you feel frustrated? | .716 | .733 | .730 | .702 | .677 | .749 | .593 | .743 | .730 | .728 | |
| 11 | Because of your tinnitus do you feel that you have a terrible disease? | .530 | .464 | .557 | .528 | .310 | .017 | .503 | .525 | .569 | .462 | |
| 12 | Does your tinnitus make it difficult for you to enjoy life? | .685 | .730 | .716 | .763 | .760 | .648 | .641 | .641 | .782 | .549 | |
| 13 | Does your tinnitus interfere with your job or household duties? | .640 | .689 | .647 | .717 | .663 | .723 | .669 | .690 | .359 | -.188 | |
| 14 | Because of your tinnitus do you find that you are often irritable? | .680 | .730 | .683 | .704 | .587 | .652 | .620 | .700 | .762 | .826 | |
| 15 | Because of your tinnitus is it difficult for you to read? | .555 | .665 | .543 | .657 | .499 | .496 | .682 | .732 | .591 | .404 | |
| 16 | Does your tinnitus make you upset? | .642 | .623 | .694 | .745 | .828 | .848 | .588 | .716 | .792 | .829 | |
| 17 | Do you feel that your tinnitus problem has placed stress on your relationship with members of your family and friends? | .667 | .676 | .677 | .693 | .757 | .604 | .572 | .650 | .586 | .190 | |
| 18 | Do you find it difficult to focus your attention away from your tinnitus and on other things? | .685 | .738 | .693 | .752 | .662 | .732 | .587 | .624 | .792 | .647 | |
| 19 | Do you feel that you have no control over your tinnitus? | .336 | .529 | .327 | .492 | .182 | -.177 | .406 | .563 | .635 | .538 | |
| 20 | Because of your tinnitus do you often feel tired? | .502 | .590 | .488 | .556 | .550 | .525 | .540 | .601 | .770 | .713 | |
| 21 | Because of your tinnitus do you feel depressed? | .715 | .754 | .726 | .768 | .659 | .577 | .612 | .660 | .772 | .713 | |
| 22 | Does your tinnitus make you feel anxious? | .596 | .557 | .610 | .630 | .556 | .661 | .549 | .589 | .693 | .664 | |
| 23 | Do you feel that you can no longer cope with your tinnitus? | .688 | .663 | .706 | .669 | .711 | .566 | .534 | .619 | .696 | .781 | |
| 24 | Does your tinnitus get worse when you are under stress? | .288 | .284 | .276 | .198 | .224 | .380 | .352 | .364 | .575 | .554 | |
| 25 | Does your tinnitus make you feel insecure? | .638 | .688 | .642 | .687 | .826 | .803 | .574 | .682 | .616 | .352 | |

BL: Baseline, FV: Final Visit, grey marked cells indicate r<0.3

Corrected item/total correlation for TBF12

| **Corrected Item/total correlation of the TBF-12 questions** | | | | | | | | | | | |
| --- | --- | --- | --- | --- | --- | --- | --- | --- | --- | --- | --- |
| **No** | **Question** | **Total** | | **German** | | **Dutch** | | **Portuguese** | | **Spanish** | |
|  |  | **BL**  **(n=685)** | **FV**  **(n=341)** | **BL**  **(n=534)** | **FV**  **(n=238)** | **BL**  **(n=35)** | **FV**  **(n=19)** | **BL**  **(n=83)** | **FV**  **(n=71)** | **BL**  **(n=33)** | **FV**  **(n=13)** |
| 1 | Because of your tinnitus is it difficult for you to concentrate? | .608 | .653 | .590 | .618 | .628 | .760 | .550 | .532 | .796 | .698 |
| 2 | Is it difficult for you to understand what people are saying because of the intensity of your tinnitus? | .443 | .563 | .427 | .477 | .592 | .686 | .382 | .627 | .672 | .708 |
| 3 | Do you get annoyed by your tinnitus? | .563 | .628 | .558 | .590 | .621 | .548 | .542 | .644 | .772 | .629 |
| 4 | Do you feel that you cannot escape your tinnitus? | .477 | .595 | .470 | .504 | .388 | .518 | .342 | .636 | .736 | .670 |
| 5 | Does your tinnitus interfere with your social activities (such as going out to dinner, to the movies)? | .619 | .670 | .609 | .611 | .579 | .510 | .655 | .727 | .560 | .000 |
| 6 | Do you feel frustrated because of your tinnitus? | .635 | .698 | .655 | .681 | .572 | .605 | .488 | .644 | .729 | .788 |
| 7 | Does your tinnitus interfere with your job or household responsibilities? | .697 | .706 | .702 | .670 | .743 | .697 | .593 | .765 | .681 | -.292 |
| 8 | Because of your tinnitus is it difficult for you to read? | .530 | .648 | .517 | .601 | .515 | .637 | .523 | .720 | .586 | .299 |
| 9 | Do you feel that your tinnitus has placed stress on your relationship with members of you family and friends? | .679 | .691 | .678 | .648 | .735 | .681 | .639 | .753 | .634 | .591 |
| 10 | Do you find it difficult to focus your attention on things other than your tinnitus? | .597 | .654 | .605 | .569 | .667 | .701 | .437 | .733 | .716 | .342 |
| 11 | Does your tinnitus make you feel anxious? | .452 | .438 | .550 | .515 | .586 | .508 | .355 | .576 | .552 | .356 |
| 12 | Do you feel that you can´t cope with your tinnitus? | .607 | .616 | .645 | .617 | .579 | .658 | .503 | .563 | .623 | .520 |

BL: Baseline, FV: Final Visit, grey marked cells indicate r<0.3

Corrected item/total correlation for TQ

| **Corrected Item/total correlation of the TQ questions* at** | | **Baseline** | **Final Visit** |
| --- | --- | --- | --- |
| **No** | **Question** | **total/German (n=983)** | **total/German (n=295)** |
| 1 | I can sometimes ignore the noise even when are there | .467 | .534 |
| 2 | I am unable to enjoy listening to music because of the noise | .510 | .609 |
| 3 | It’s unfair that I have to suffer with my noise | .460 | .519 |
| 4^†^ | I wake up more in the night because of my noise | .558 | .526 |
| 5^‡^ | I am aware of the noise from the moment I get up to the moment I sleep | .534 | .505 |
| 7 | Most of the time the noise are fairly quiet | .461 | .528 |
| 8 | I worry that the noise will give me a nervous breakdown | .571 | .582 |
| 9^‡^ | Because of the noise I have difficulty in telling where sounds are coming from | .395 | .461 |
| 10 | The way the noise sounds is really unpleasant | .529 | .605 |
| 11^†^ | I feel I can never get away from the noise | .553 | .601 |
| 12 | Because of the noise I wake up earlier in the morning | .554 | .558 |
| 13 | I worry where I will able to put up with this problem for ever | .661 | .695 |
| 14 | Because of the noise it is more difficult to listen to several people at once | .420 | .507 |
| 15^†^ | The noise are loud most of the time | .528 | .615 |
| 16 | Because of the noise I worry that there is something seriously wrong with my body | .453 | .543 |
| 17^†‡^ | If the noise continue my life will not be worth living | .657 | .662 |
| 18 | I have lost some of my confidence because of the noise | .644 | .644 |
| 19 | I wish someone understood what this problem is like | .565 | .560 |
| 20 | The noise distract me whatever I am doing | .619 | .675 |
| 21 | There is very little one can do to cope with the noise | .515 | .570 |
| 22 | The noise sometimes give me a pain in the ear or head | .484 | .459 |
| 24^†‡^ | I am more irritable with my family and friends because of the noises | - | - |
| 25 | Because of the noises I have tension in the muscles of my head and neck | .414 | .344 |
| 26 | Because of the noises I have other peoples voices sound distorted to me | .432 | .563 |
| 27 | It will be dreadful if these noises never go away | .638 | .655 |
| 28^‡^ | I worry that the noises might damage my physical health | .578 | .595 |
| 31 | Sleep is my main problem | .435 | .372 |
| 33 | I have more difficulty following a conversation because of the noises | .501 | .557 |
| 34^†‡^ | I find it harder to relax because of the noises | .643 | .695 |
| 35^†‡^ | My noises are often so bad that I cannot ignore them | .569 | .640 |
| 36^‡^ | It takes me longer to get to sleep because of the noises | .560 | .475 |
| 37 | I sometimes get very angry when I think about having the noises | .573 | .641 |
| 38 | I find it harder to use the telephone because of the noises | .513 | .524 |
| 39^†‡^ | I am more liable to feel low because of the noises | .662 | .692 |
| 41 | Because of the noises life seems to be getting on top of me | .657 | .621 |
| 43^‡^ | I often think about whether the noises will ever go away | .519 | .561 |
| 44 | I can imagine coping with the noises | .466 | .516 |
| 47^†‡^ | I am a victim of my noises | .652 | .653 |
| 48^†^ | The noises have affected my concentration | .635 | .617 |
| 50 | Because of the noises I am unable to enjoy the radio or television | .540 | .623 |
| 51 | The noises sometimes produce a bad headache | .430 | .434 |

* analysis only of the 40 questions used for calculation of total score (item 24 listed because it is part of Mini-TQ and TQ 12)
^†^ item of Mini-TQ, ^‡^ item of TQ 12
grey marked cells indicate r<0.3
